# The tempo of human childhood: a maternal foot on the accelerator, a paternal foot on the brake

**DOI:** 10.1002/evan.21579

**Published:** 2018-03-25

**Authors:** Jennifer Kotler, David Haig

**Affiliations:** ^1^ Harvard University Department of Organismic & Evolutionary Biology

**Keywords:** genomic imprinting, parent‐offspring conflict, adrenarche, puberty, weaning

## Abstract

Relative to the life history of other great apes, that of humans is characterized by early weaning and short interbirth intervals (IBIs). We propose that in modern humans, birth until adrenarche, or the rise in adrenal androgens, developmentally corresponds to the period from birth until weaning in great apes and ancestral hominins. According to this hypothesis, humans achieved short IBIs by subdividing ancestral infancy into a nurseling phase, during which offspring fed at the breast, and a weanling phase, during which offspring fed specially prepared foods. Imprinted genes influence the timing of human weaning and adrenarche, with paternally expressed genes promoting delays in childhood maturation and maternally expressed genes promoting accelerated maturation. These observations suggest that the tempo of human development has been shaped by consequences for the fitness of kin, with faster development increasing maternal fitness at a cost to child fitness. The effects of imprinted genes suggest that the duration of the juvenile period (adrenarche until puberty) has also been shaped by evolutionary conflicts within the family.

## 1 INTRODUCTION

Theories of life‐history evolution typically assume that natural selection maximizes an individual's fitness, but natural selection does not maximize individual fitness when individuals interact with kin. One must also consider the effects of changes in an individual's life history on the fitness of relatives. If delayed maturation has costs to kin, then natural selection would favor earlier maturation than would be predicted by considerations of individual fitness alone. Conversely, if delayed maturation benefits kin at a cost to individual fitness, then natural selection would favor later maturation than maximizes individual fitness. But one cannot simply replace individual fitness with inclusive fitness and maximize the latter because inclusive fitness is calculated differently for each member of the family. Consider the evolution of interbirth intervals (IBIs); that is, how long postpartum a mother remains nonpregnant. A mother's lifetime fecundity (her number of surviving offspring) will have benefited from shorter IBIs than maximize each individual child's chances of survival. Therefore, the duration of postpartum infertility will have been evolutionarily contested, with infants evolving to act in ways that marginally increase the time until the arrival of a younger sibling, for example by increased intensity of suckling and increased waking at night.^1^ Since actions of mothers and infants both influence the duration of IBIs, the evolutionary outcome of this conflict need not maximize the inclusive fitness of either party. Similar conflicts exist over optimal birth weights,^2^ the tempo of childhood maturation, and optimal timing of puberty.^3^, ^4^

Inclusive fitness was classically defined as the property of an individual that was the target of natural selection in interactions among relatives who share some of their genes but not others. Intragenomic conflicts arise when the shared and unshared fractions of interactants' genomes benefit from different outcomes of an interaction. In such cases, one can define different inclusive fitnesses for different genes within a genome.^5^ In particular, an individual's genes of maternal and paternal origin have different relatedness to matrilineal and patrilineal kin and therefore have different genic fitnesses in interactions with these kin. This intragenomic conflict is expressed in the phenomenon of genomic imprinting, whereby a gene is expressed differently when inherited from mothers or fathers (Box 1).

Box 1 **Genomic imprinting**

1 Genomic imprints mark the subset of genes for which expression depends on the sex of the parent from whom the gene was inherited. The imprint is a record of a gene's sex‐of‐origin in the previous generation. Insulin‐like growth factor 2 (*IGF2*) can serve as an example. *IGF2* is an autosomal gene that is expressed when inherited from an individual's father but not when inherited from an individual's mother.^100^ A differentially methylated region (DMR) associated with *IGF2* undergoes cytosine methylation when it passes through a male germline but not when it passes through a female germline. The methylated or unmethylated state of the DMR, which is inherited by offspring and stably maintained in somatic cells, determines which copy of the gene is expressed but is reset in the offspring's germ cells.^101^ Thus, the methylated DMR that an offspring inherits from its father and the unmethylated DMR that the offspring inherits from its mother both become demethylated in germ cells if the offspring is female, whereas both become methylated in germ cells if the offspring is male.

**Figure 5:**
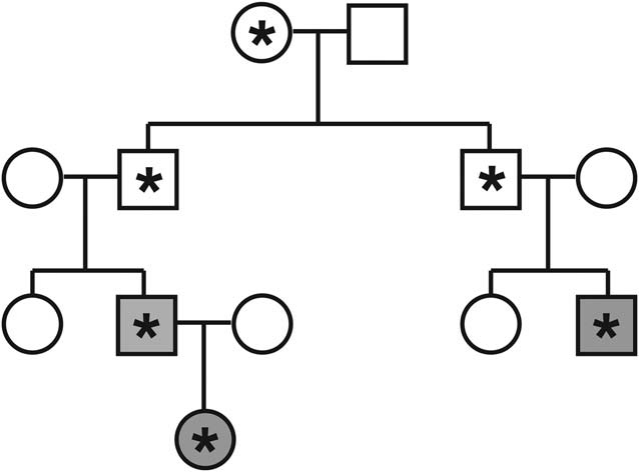
Asterisks represent an inactivating mutation in imprinted gene IGF2. In typically developing individuals, IGF2 is expressed only when inherited from the male parent. While the mutation can be inherited from either parent, only individuals who inherit the mutation from their fathers display the disease phenotype. Those who receive the mutated IGF2 allele from their mothers express only the nonmutant copy inherited from their fathers and therefore show typical development.

Some consequences of genomic imprinting can be illustrated by a multigenerational family that segregated for an inactivating mutation of *IGF2* and for intrauterine growth retardation (Figure 5). Individuals who inherited the mutation from their mothers underwent normal fetal growth because the mutant gene copy was silenced by imprinting, but the nonmutant copy inherited from their fathers was expressed. In contrast, individuals who inherited the mutation from their fathers suffered intrauterine growth retardation because their paternal gene copies were inactivated by mutation and their maternal gene copies by their unmethylated DMRs.

Over 315 imprinted genes have been identified in the human genome, with effects in more than 45 tissue types.^102^ At first sight, the existence of imprinting poses an evolutionary puzzle because the individual foregoes the advantages of having a back‐up gene copy in case one gene copy is mutated. Given that an individual's maternal and paternal gene copies are equally likely to be transmitted to the individual's descendants, what advantage to an imprinted gene could justify this cost? This paradox can be resolved by recognizing that a gene's expression in one individual can have fitness consequences for nondescendant kin with different probabilities of carrying copies of the individual's maternal and paternal alleles.^103^

Hamilton's Rule predicts that a behavior will be favored by natural selection if *rB* > *C* where *B* is the benefit of the behavior to the recipient, *C* is the cost of the behavior to the actor, and *r* is the probability that a gene responsible for the actor's behavior has an identical‐by‐descent copy in the recipient. Consider a behavior performed by an offspring that provides benefit *B* to its mother at cost *C* to the offspring's own fitness and that is caused by an imprinted (MEG). A MEG has effects only when inherited from mothers, in which case *r*_m_ = 1. Therefore, the behavior is favored by natural selection if *B* > *C*. If, on the other hand, the behavior is caused by an imprinted PEG, then the coefficient of relatedness of the child's paternal gene to the mother is *r*_p_ = 0 and the costly behavior is opposed by natural selection, no matter how great the benefit to the mother. An unimprinted gene is constrained to have the same effects when it is inherited from mothers and fathers. Therefore, an unimprinted gene that causes offspring to perform the behavior will be favored, on average, when *rB* > *C* where *r* = (*r*_m_ + *r*_p_)/2 = ½. Thus, Hamilton's rule has different forms for unimprinted genes, MEGs, and PEGs. For simplicity, our analysis assumes that a benefit to the mother is not associated with an indirect benefit to the offspring's father, as occurs when females have multiple offspring with the same partner. However, relaxation of this assumption does not affect the qualitative predictions that MEGs of offspring should reduce demands on mothers (or the mother's kin) or increase demands on fathers (or the father's kin), whereas PEGs of offspring are predicted to have the opposite effects. As a corollary, effects of imprinted genes on a life‐history trait provide strong evidence that the trait has been subject to evolutionary conflicts within the family.

Human childhood has been conceptually divided into three major phases of progressively greater independence from adults^3^, ^6^, ^7^, ^8^, ^9^: a nurseling phase from birth to weaning, a weanling phase from weaning to adrenarche; and a juvenile phase from adrenarche to gonadarche. In this review, we refer to offspring in the youngest category as “nurselings” rather than “infants” because we will argue that the definition of infancy in great apes (birth to weaning) corresponds to the combined nurseling and weanling phases of humans. Similarly, we use “weanling” in preference to “toddler” or “child” because toddler is used for a narrower age range and child is commonly used for the entire period from birth to adolescence. We review evidence of an important role of imprinted genes in the timing of weaning, adrenarche, and gonadarche. These effects of imprinted genes strongly suggest that the durations of nurseling, weanling, and juvenile phases have been subject to intergenerational and intragenomic conflicts mediated by costs and benefits for kin. Moreover, the direction of effects of maternally expressed genes (MEGs) and paternally expressed genes (PEGs) provide clues to the nature of these evolutionary trade‐offs.

## 2 HUMAN CHILDHOOD IN COMPARATIVE PERSPECTIVE

Eruption of the first permanent molar (M1) occurs around the age of weaning in many primates,^10^ but in great apes occurs well before weaning to accommodate an extended period in which offspring consume solid food as well as milk.^11^ For example, M1 of orangutans erupts at 4 years of age, but weaning occurs after 7 years. Humans are highly atypical: infants are weaned at 2–3 years of age but M1 does not emerge until 6 years or thereabouts.^12^ Thus, weaning has been accelerated in the human lineage relative to our closest relatives, but there has been no corresponding acceleration in the tempo of molar eruption. As a consequence, human weanlings must subsist on specially prepared foods because their deciduous dentition is unable to process an adult diet.^13^

**Figure 1:**
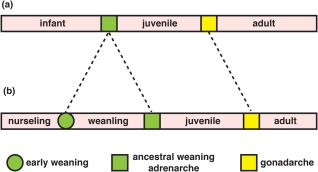
(A) For most great apes, the progression from birth to sexual maturation can be broken into three major stages. However, we describe human maturation as occurring over four stages (B). This has evolved by the separation of weaning and adrenarche into two distinct processes. There is significant variation in the ages at which each stage is met, but a rough estimate would be: nurseling (birth‐2.5 years), weanling (2.5‐7 years), juvenile (7 years‐13 years), adult (puberty onward) [Color figure can be viewed at http://wileyonlinelibrary.com]

Chimpanzees, gorillas, and orangutans all nurse their infants until they become nutritionally and socially independent juveniles^14^ (Table 1). Thus, these mothers intensively care for only one offspring at a time and their IBIs approximate the age of juvenile independence. The interpolation of a weanling phase into human life history, during which weaned offspring subsist on a special diet, has been considered a key evolutionary innovation because it enabled short IBIs to coexist with prolonged childhoods (Figure 1). However, this innovation required that mothers or their surrogates simultaneously provision both a nurseling with milk and a weanling with specially prepared foods. Current estimates vary, but caregivers generally begin to supplement a child's diet when the child is about 18 months old and continue to introduce foods until roughly the age of adrenarche (Box 2).^15^ The standard interpretation of how this was possible is that women had support from other group members, who contributed time and resources to the care of mothers and their offspring.^16^, ^17^

**Table 1:** Age at adrenarche, weaning and average interbirth interval (IBI) for great apes (all ages in years)^a^

Species	Age at First Birth^118^	Age at Adrenarche	Age at Weaning	Average IBI
**Bonobos**	14.2	5	4‐5	4‐6
**Chimpanzee**	13.3	5	5	5
**Gorillas**	10.0	5	3‐4	3‐4
**Orangutans**	15.6	8	7‐8	7‐8
**Humans**	19.5	5‐8	2‐3	3.7^119^

a Adrenarche and weaning are aligned in each species with the exception of humans. These data suggest that adrenarche may play a role in the preparation for behavioral independence at weaning in juvenile apes. The separation of weaning and adrenarche in humans reduces the IBI, giving the human mother an opportunity to fit two births into the IBI of other great apes.

Box 2 **Adrenarche**

1 Androgen production by the adrenal cortex varies markedly with age. During pregnancy, the fetal adrenal cortex is a major source of dehydroepiandrosterone (DHEA) and its sulfate (DHEAS). These weakly androgenic steroids are aromatized by the placenta to estrogens that are secreted into the maternal circulation. The “fetal zone” of the adrenal cortex regresses soon after birth, with DHEAS falling to undetectable levels in infants.^104^ Secretion of DHEAS resumes from the newly differentiated zona reticularis of the adrenal cortex later in childhood.^105^ DHEAS is detectable as early as three years of age in some children,^106^, ^107^ with levels rising to a peak at 24–30 years, then gradually decline into old age.^108^ The physiological functions of these long‐term changes in adrenal androgens are poorly understood.

Although low levels of adrenal androgens can be detected as early as 3 years, adrenarche is usually said to occur at 5–8 years, when serum DHEAS reaches levels readily detectable by standard techniques.^109^ This increase of DHEAS in mid‐childhood has been termed “biochemical adrenarche,” whereas outward signs of androgenic activity such as pubic and axillary hair, adult body odor, and oily skin have been termed “clinical adrenarche.'^110^ The phenotypic manifestations of clinical adrenarche probably are responses to the conversion of DHEA to more active androgens in target tissues.^111^ DHEA is also believed to have endocrine functions that are independent of its conversion to potent sex steroids.^112^ Clinical adrenarche is described as premature if it occurs before 8 years of age in girls and 9 years in boys.^109^ Premature adrenarche could be caused by either an early onset of adrenal androgen synthesis, with a subsequent “normal” rate of increase, or an onset at the same age but with accelerated increase in production. Either scenario would result in biochemical and clinical thresholds being reached at younger ages.

Adrenarche is often conceptualized as a stage of sexual maturation because pubic and axillary hair are considered sexual characters and activation of gonadal steroidogenesis (gonadarche) regularly follows adrenarche. From this perspective, the physical manifestations of clinical adrenarche are ascribed to early stages of puberty.^109^ But adrenarche is neither necessary nor sufficient for progression to sexual maturity. Children can undergo adrenarche without subsequent gonadarche or gonadarche without prior adrenarche.^113^, ^114^ We suggest that it may be more fruitful to think of adrenarche as a gradual process that is part of social, rather than sexual, maturation. The resumption of adrenal androgen synthesis from the zona reticularis at about 3 years of age roughly coincides with the age of weaning in natural fertility populations and the timing of clinically premature adrenarche.^115^ “Biochemical adrenarche” coincides with the transition to middle childhood^116^ and the cognitive and emotional changes associated with greater independence and responsibility that have been characterized as the “five‐to‐seven‐year shift.”^117^ Thus, biochemical adrenarche is a hormonal correlate, and possibly a contributing cause, of these behavioral changes, with clinical adrenarche serving as a phenotypic marker of these changes.^6^, ^8^

Hormonal changes also occur as primates undergo these nutritional shifts. Adrenal androgens increase at about 5 years of age in semi‐captive bonobos, chimpanzees, and gorillas^18^, ^19^ and at about 8 years of age in orangutans,^20^ roughly corresponding with ages at weaning: 7–8 years in orangutans and 3–5 years in the other great apes (Table 1). This correlation, based on limited data, suggests that adrenal androgens may play a role in the increased independence observed in juvenile apes at weaning.^14^, ^21^ Despite significant variation in adrenarcheal timing,^22^ adrenarche in humans is considered to occur at 5–8 yrs of age, (Box 2), long after weaning and at about the age of molar eruption and shift to an adult diet. Because data on adrenal maturation in apes are sparse,^14^, ^23^ the hypothesis that adrenarche is a hormonal correlate of weaning in great apes and, by implication, in chimpanzee‐human ancestors, must remain a conjecture until reliable longitudinal data on changes in adrenal androgens are available for nonhuman primates.

**Figure 2:**
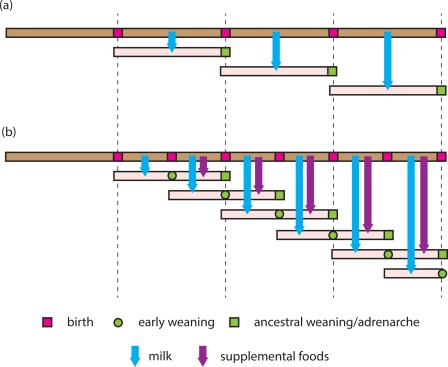
(A) The life history of an ancestral hominin female is proposed to resemble that of the other great apes. A female nurses an infant until it becomes quasi‐independent (coinciding with adrenarche). It is only after this child is capable of self‐support that the female is able to nurse her next offspring. (B) The modern human life history involves early weaning onto a supplemental diet before an offspring is self‐supporting, thus freeing the mother to be able to nurse a younger sibling. A mother may thus be simultaneously engaged in the intensive support of two offspring, providing the younger with milk and the elder with supplemental foods. In the stylized version of this life history, it is not until the elder child goes through adrenarche and the younger child is weaned that the mother is free to nurse her next child. The intensive pattern of reproduction by human females was made possible by other group members providing the mother with nutritional support during child care [Color figure can be viewed at http://wileyonlinelibrary.com]

The pattern of adrenarcheal timing and dental eruption in humans and great apes can be brought into a coherent framework if the mid‐childhood transition in humans, associated with molar eruption and biochemical adrenarche, is considered to be homologous to the transition to greater independence of juvenile apes at weaning. Human weaning can then be considered to have been dissociated into two phases, the transition from milk to a special diet at about 3 years and the gradual but complete transition to an adult diet by about 7 years.^15^ From this perspective, the prolonged infancy of great apes corresponds to the combined nurseling and weanling phases of human development (Figure 2).

**Figure 3:**
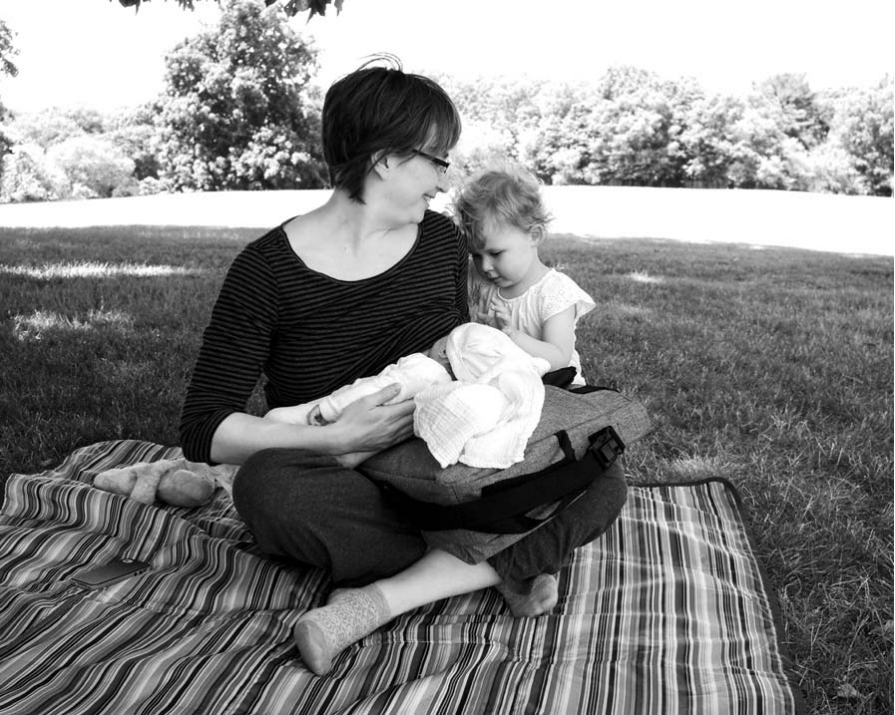
Unlike other apes, humans are able to concurrently care for both a nurseling and a weanling

While adrenarche occurs at similar ages in orangutans and humans, the average human IBI is less than half that of orangutans.^12^ The combined duration of nurseling and weanling phases (birth to adrenarche) equals roughly two human IBIs: when a mother becomes pregnant with her next child, her youngest child is being weaned and her next youngest child is starting to provide for itself (Fig 1b). Human mothers have thus been responsible for the intensive care of two offspring, a weanling and a nurseling, at one time (Figure 3). Supplemental feeding of weanlings allows human mothers to double their effective fecundity relative to that of their closest simian relatives.

Human mothers invest substantial time in social interaction with nurselings and weanlings. Changes in a child's social behavior as it enters the juvenile phase partially relieve mothers of this attentional burden. Cross‐culturally, middle childhood is marked by a shift in social engagement from a primary focus on mothers to increased interaction with peers.^6^ Middle childhood also corresponds to the start of full‐time schooling in most developed nations. The first day of school is often the first time that a child spends extended time apart from its mother. In traditional societies, this is also the age at which offspring begin to engage in economic work.^16^, ^24^ These transitions require the formation of social bonds outside the family, with the child assuming responsibility for maintaining these relations and thereby facilitating social and cognitive development beyond what the mother alone is able to provide.^8^

Ancestral human juveniles would have exhibited increasing independence as they shifted from exclusive reliance on parents or alloparents to foraging for themselves and partaking in broader community resources.^3^ Evolutionary theorists have previously considered the participation of juveniles as helpers in human cooperative breeding.^24^, ^25^ By the ages of 5 to 7 years, children from modern natural‐fertility populations typically produce about half of what they consume.^16^ Such juvenile “foraging” would have significantly reduced the overall burden of maternal investment in caring for multiple offspring. Children of this age share food more equitably within a group than do younger children^26^ and show an in‐group sharing bias, as one would expect from contributing members of the family unit.^26^ Ancestral communal groups were likely to have included full sibs and maternal half sibs. Therefore, the willingness of juveniles to redistribute resources would have further benefited maternal fitness by contributing to the care of matrilineal sibs.^4^

Human sexual maturation is generally considered to be delayed relative to that of great apes (Table 1), but there is substantial variation within and between human populations.^27^, ^28^ Estimates of heritability indicate substantial genetic and environmental contributions to within‐population variation in age of puberty.^29^ Both sources of variation raise interesting evolutionary questions. Does some form of frequency‐dependent selection maintain genetic variation for age at puberty? How much of the environmental variability is nonadaptive “noise” and how much adaptive responses to environmental cues?

## 3 INTERGENERATIONAL AND INTRAGENOMIC CONFLICTS OF NURSELING AND WEANLING PHASES

Women in natural‐fertility populations are infertile while nursing (lactational amenorrhea). Weaning occurs on their return to fertility, sometimes when the mother is already pregnant. Thus, there is a strong correlation between the age at weaning and the IBI. Maternal fecundity would be increased by earlier weaning because this would enable mothers to fit more births into a given period, but the probability of child death or morbidity would also increase. Evolutionary theory predicts intergenerational conflict between mothers and offspring over the timing of weaning^30^ and intragenomic conflict within offspring genomes between genes of maternal and paternal origin.^31^ Similar conflicts are expected over the age of adrenarche, with earlier adrenarche allowing a mother to concentrate her care on a younger infant. In this section, we discuss evidence that PEGs favor prolongation of the nurseling and weanling phases, whereas MEGs favor the reverse.

**Figure 4:**
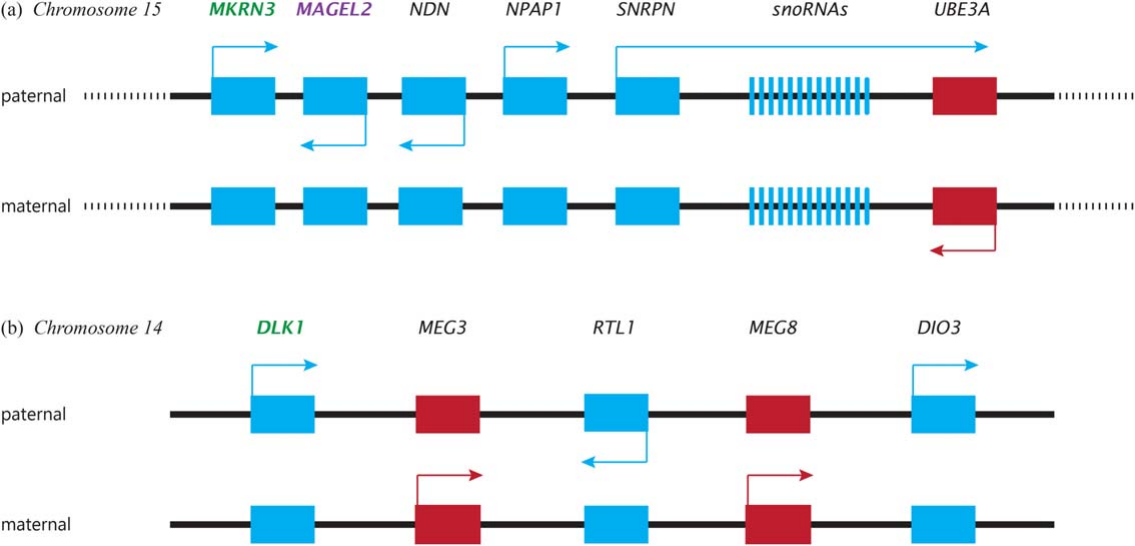
A simplified map of the cluster of imprinted genes on human chromosome 15 and 14. Red alleles are MEGs, blue alleles are PEGs. Green labels indicate known puberty promoters Purple label denotes known pubertal inhibitor. (A) Chromosome 15: The gene cluster is flanked by repeated sequences (represented by dotted lines). Recombination between the repeats in paternal meiosis results in deletion of the paternal copy of the cluster and a diagnosis of Prader–Willi syndrome. Recombination in maternal meiosis results in deletion of the maternal copy of the cluster and a diagnosis of Angelman syndrome. Mutations of the paternal copy of *MKRN3* result in precocious puberty, whereas mutations of the paternal copy of *MAGEL2* or deletions of the paternal cluster of small nucleolar RNAs (snoRNAs) results in hypogonadism and incomplete puberty. Mutations of the maternal copy of *UBE3A* are a cause of familial Angelman syndrome. (B) Chromosome 14: Temple syndrome (TS14) is caused by uniparental maternal expression of genes in this region. *DLK1* mutations are associated with precocious puberty. Genetic variants in *DLK1* are associated with menarche timing in girls and age at voice‐breaking in boys. Other alleles in this region are responsible for characteristics traditionally associated with kinship conflict in genomic imprinting, including maternal behaviors (*MEG3/GTL2)* and nonshivering thermogenesis in newborn mice (*DIO3)* [Color figure can be viewed at http://wileyonlinelibrary.com]

Prader–Willi syndrome (PWS) and Angelman syndrome (AS) have attracted particular attention as human disorders of imprinted genes with distinctive phenotypes in childhood.^21^, ^32^, ^33^ Most cases of PWS are caused by deletion of a cluster of imprinted genes on the paternally inherited copy of chromosome 15 (paternal deletion) or by inheritance of both copies of this cluster from the father (paternal uniparental disomy) (Figure 4). Therefore, the characteristic phenotype of PWS can be ascribed to the absence of expression of PEGs. Maternal deletion or maternal uniparental disomy of the same gene cluster results in AS with symptoms that can be ascribed to absence of expression of MEGs. Infants with PWS exhibit weak suck, disinterest in feeding, and excessive sleepiness, whereas infants with AS exhibit excessive wakefulness. An evolutionary interpretation is that PEGs, which are absent in PWS, were selected to favor intense suckling and frequent waking to prolong lactational amenorrhea and delay weaning. In contrast, MEGs, which are absent in AS, were selected to favor less frequent waking, earlier weaning, and shorter IBIs. The behavior of typically developing children, who inherit copies of the gene cluster from both parents, is determined by the balance of effects of PEGs and MEGs. When new mothers complain of exhaustion, their fatigue can be considered an adaption or extended phenotype of genes that the baby inherits from its father.^1^

Effects of imprinted genes on the age of adrenarche have been less studied, in part because adrenarche itself has been less studied. Nevertheless, children with PWS produce more adrenal androgens than do typically developing children from three years of age.^34^ Thus, PEGs on chromosome 15 appear to inhibit the production of adrenal androgens, prolonging the weanling phase and the duration of dependence on mothers. The absence of expression of these genes in PWS causes threshold levels of adrenal androgens to be exceeded at younger ages and to be clinically expressed as precocious adrenarche. Silver Russell Syndrome (SRS) is another imprinting disorder caused by uniparental maternal expression of alleles on either chromosome 7 or 11. Patients with SRS are similar to those with PWS in that they more likely than members of the average population to undergo early or premature adrenarche.^35^ Further investigation in SRS and other imprinting disorders will test this association between PEGs and adrenarcheal inhibition.

PWS is associated with characteristic age‐related changes in appetite in which neonatal anorexia (poor suck and disinterest in feeding) flips in later childhood to excessive eating and obsession with food. This phenotypic progression was initially interpreted as the outcome of evolutionary conflicts associated with weaning and the transition from breast milk to supplemental foods.^32^, ^33^ However, we now consider the complex changes in appetite and food‐related behaviors in PWS to reflect evolutionary conflicts associated with the extended “weaning” of human development, first from the breast and then from supplemental foods.^21^ In this revised scheme of feeding behaviors, anorexia in nurselings with PWS changes to improved appetite in weanlings, with the onset of extreme hyperphagia and “foraging” delayed until the beginning of the juvenile phase after early adrenarche.

## 4 IMPRINTING EFFECTS ON PUBERTAL TIMING

The juvenile phase begins with adrenarche and ends with gonadarche, which is commonly referred to as puberty, although the latter term is sometimes used to include both gonadarche and adrenarche (Fig 2). On average, gonadarche occurs at a younger age in girls (8–13 years) than boys (9.5–13.5 years).^36^ Some genetic effects on the timing of human puberty are sex‐independent; others are sex‐specific. Many genetic variants associated with age at menarche are also associated with age at voice breaking in males with the same direction of effects.^37^ As a consequence, early‐maturing girls tend to have early‐maturing brothers and late‐maturing boys tend to have late‐maturing sisters. While clinical understanding of the role imprinted genes play in pubertal timing is still developing, here we consider preliminary evidence of the effects of imprinted genes on pubertal timing; in the next section, we consider how the timing of puberty may have affected the fitness of relatives.

Central precocious puberty is clinically defined as the presence of secondary sexual characteristics before the age of 8 years in girls and 9 years in boys.^38^ About half of all cases of familial central precocious puberty carry a mutated paternal copy of *MKRN3*, a PEG from the PWS/AS region of chromosome 15.^38^ Furthermore, most children with uniparental maternal disomy of chromosome 14, known as Temple syndrome, or TS14, undergo precocious puberty.^39^ *DLK1* is included in a cluster of imprinted genes on chromosome 14 (Fig 4). This gene was recently found to be mutated in four female relatives who experienced precocious puberty, all of whom inherited the mutated *DLK1* allele from their father.^40^ In genome‐wide association studies, paternally inherited variants at *MKRN3* and *DLK1* are correlated with age at menarche in girls and voice‐breaking in boys.^37^, ^41^ These findings suggest that expression of *MKRN3* and *DLK1*, both PEGs, inhibits pubertal progression — puberty occurs at a younger age if either gene is inactive — and that genetic variation at or near these loci is associated with normal variation in the age of puberty. Such evidence of the important role imprinted genes play on pubertal timing suggests that the tempo of sexual maturation had evolutionarily significant effects on the fitness of a child's kin and that these effects were differently experienced by kin from the maternal versus paternal line (see Box 1).

The association of inactivating mutations of *MKRN3* with precocious puberty suggests that expression of *MKRN3* promotes pubertal delay. Occasional individuals with PWS undergo precocious puberty,^42^, ^43^ but most undergo incomplete puberty, expressed as lack of a pubertal growth spurt, hypogonadotropic hypogonadism, cryptorchidism, underdeveloped genitalia, and incomplete menarche.^44^, ^45^, ^46^, ^47^, ^48^, ^49^ Thus, other PEGs from this chromosomal region appear to promote, rather than inhibit, aspects of sexual maturation. Most males with PWS exhibit primary testicular failure that becomes apparent after puberty.^49^ A careful study of a group of boys with PWS found that early stages of puberty were accelerated, but pubertal progression arrested abruptly in mid‐puberty.^50^ Female patients exhibit impaired maturation of ovarian follicles.^48^

The rarity of precocious puberty in PWS, despite the absence of expression of *MKRN3*, is probably explained by the effects of other imprinted genes that are inactivated in typical cases of PWS (Fig 4). Mutations in *MAGEL2*, a close neighbor of *MKRN3*, are associated with undescended testes and/or a micropenis in most boys.^51^ In a girl, a small deletion that eliminated paternal copies of *MKRN3* and *MAGEL2* but spared most other genes that are typically deleted in PWS was associated with pubic hair and breast development at 7.5 years, along with accelerated bone age but without typical features of PWS.^52^ In contrast, deletions that spare *MKRN3* and *MAGEL2* but remove nearby paternally expressed small nucleolar RNAs (snoRNAs) are associated with hypogonadism and typical features of PWS.^53^ These contrasting phenotypes suggest that effects of nonexpression of *MKRN3* on pubertal timing are obscured in typical cases of PWS by the effects of nonexpression of other PEGs having the usual function of promoting gonadal maturation and steroidogenesis. The effect of *MKRN3* mutations on pubertal timing are more pronounced in girls than boys.^38^, ^54^, ^55^ It is possible that the effects of imprinted genes later in life are simply byproducts of their adaptive value in early development. However, given the complex mechanistic systems at play for imprinted genes, in which a given gene's activation can be specific both to tissue type and developmental stage,^56^, ^57^ the robust effects of imprinted genes on the timing of gonadarche suggests a more substantial adaptive role for these genes. The complex pubertal phenotype of PWS, in which some aspects of maturation are accelerated and others suppressed, suggests that effects of the pubertal transition on the fitness of kin were similarly complex. Natural selection will have acted on when to become an adult and on what kind of adult to become.

Unlike the ambiguous picture in PWS, individuals with TS14 clearly exhibit central precocious puberty with accelerated bone age, increased height velocity, early breast development, early menarche, and pubertal levels of reproductive hormones.^58^, ^59^ In cases of TS14 associated with microdeletions, *DLK1* is the only gene in the minimal region of overlap, strongly suggesting that absence of expression of *DLK1* is responsible for precocious puberty.^59^ In a recent report, deletion of *DLK1* was associated with premature pubarche and thelarche accompanied by accelerated growth and advanced bone age in four girls who inherited the deletion from their unaffected fathers. Pubertal development was arrested with long‐acting GnRH‐agonists in these girls, but their paternal grandmother, from whom they inherited the deletion, recalled that her menarche had occurred when she was 9–10 years old.^40^

## 5 KIN CONSIDERATIONS IN PUBERTAL TIMING

Both MKRN3 and DLK1are paternally expressed imprinted genes. Therefore, the observations we have discussed suggest that earlier sexual maturation of ancestral hominins was either costly to patrilineal kin but increased individual fitness or benefited matrilineal kin but decreased individual fitness.^9^, ^60^ Here we discuss complex evolutionary trade‐offs that may contribute to imprinted expression of genes affecting the timing of pubertal onset.

Other things being equal, earlier reproduction is better than later reproduction because an individual might not survive to reproduce at the older age. Reproductive postponement is favored if delay confers future benefits that more than compensate for the risk.^61^ Although the evolution of prolonged human childhood has been widely discussed,^7^, ^24^, ^62^, ^63^ a fully satisfactory evolutionary understanding of age at sexual maturity would need to account for substantial variation of timing within human populations; the generally earlier age of puberty and greater variability of timing for girls than for boys; the occurrence of a subset of girls with very early puberty; and the direction of effects of imprinted genes. In this section, we will raise, but not answer, two interrelated questions that bear on the effects of imprinted genes. First, was the prolongation of human childhood “altruistic,” benefiting kin at a cost to individual fitness, or “selfish.” benefiting individual fitness at a cost to kin? Second, were benefits or costs to kin preferentially experienced by the maternal or paternal line?

Whether delayed puberty of her offspring would enhance or reduce a mother's fitness depends on the direct effects of pubertal timing on the fitness of each child and the extent to which juveniles are a drain on maternal investment (“another mouth to feed'” or active contributors to the care of siblings (“helpers at the nest”). The juvenile stage will have been a period of negotiation not only between mothers and juveniles, but between maternal and paternal genes within juvenile genomes over the balance between helping behaviors, favored by maternally derived genes, and self‐directed behaviors, favored by paternally derived genes. This can be conceptualized as tension between juvenile “work” and “play.”

Because patterns of dispersal would have influenced opportunities for intergenerational collaboration and competition, a further consideration is how often, after puberty, children moved away from or remained with their mother and other kin,. Reproductive competition between mothers and sexually mature daughters, including conflicts over who was to help raise whose offspring, was probably a minor factor because of minimal overlap in fertility occasioned by menopause, especially in societies where daughters left their parental homes to move into their husbands' households.^64^

There has been much discussion of proximate mechanisms of variation in pubertal timing in response to environmental cues.^28^, ^65^, ^66^, ^67^ Anthropological evidence that older sibs enhance the fitness of younger sibs is consistent with “helping at the nest.”^68^, ^69^ In preindustrial Finland, the presence of older siblings increased the probability that a child survived to sexual maturity.^70^ Nepalese girls with two or more siblings reach menarche later than girls with fewer than two siblings.^71^ The latter observation is consistent with an evolved delay in maturation to provide help, but could also be explained by slower development in larger sibships because of greater competition for resources. Eldest daughters have been claimed to reach menarche at an older age than do youngest daughters, although support for this claim has been mixed.^65^, ^72^ The absence of a father, presence of half‐brothers and step‐brothers, and measures of paternal warmth have all been associated with faster developmental trajectories in girls, including earlier age at menarche and younger age at first birth.^65^, ^72^, ^73^ These factors are potential cues of lower relatedness to younger children within the household,^74^ resulting in reduced contributions to the daughter's inclusive fitness of “helping at the nest”: care of maternal half‐sibs with different fathers provides no genetic benefit to a child's paternal genes.

The balance of selective forces may differ between the sexes. Anthropological case studies have found that daughters preferentially help mothers with child care and domestic activities,^75^ whereas sons tend to work outside the household.^76^ In fact, across primates there is only one example of young males consistently participating in child‐care duties.^77^ Therefore, the labor of boys and girls may affect different sets of kin. Compared to mothers with elder sons, mothers with elder daughters have more children in total and more children later in life.^75^, ^78^ Although mothers with elder sons and mothers with elder daughters did not differ significantly in the number of children they had in their first ten childbearing years, mothers with elder daughters produced more children in the subsequent fifteen years, coinciding with the time at which an elder daughter could provide competent child care.^78^ However, sociological factors such as a preference for sons may also play a role^79^.

## 6 FAST FEMALE LIFE‐HISTORIES?

Life‐history theory predicts that the tempo of childhood maturation should be sensitive to cues of costs and benefits of earlier versus later reproduction. In small‐scale societies, better conditions typically are associated with faster growth and earlier puberty.^80^ This association parallels dramatic increases in height and reductions in age of menarche over the past two centuries in large‐scale societies.^27^ These secular trends have been attributed to improved nutrition and escape from disease. On the other hand, girls in developed nations have been proposed to undergo earlier maturation under stressful, high‐mortality conditions.^80^, ^81^ In simple evolutionary models, the optimal age at sexual maturation will occur when the marginal benefit of delay equals the marginal cost of delay. It would not be difficult to construct a model predicting that girls with better prospects should reproduce earlier because they have increased benefits of early reproduction or that girls with poorer prospects should reproduce earlier because of increased costs of delay. Such models need not be strict alternatives because each could apply to a different segment of the population. Here we briefly consider evidence that a subset of human females adopt an accelerated life history with possible health costs at older age.

Premature adrenarche is about nine times more common in girls than boys^82^; central precocious puberty is 15–20 times more common.^83^ Most cases of central precocious puberty in girls are without obvious pathology and could plausibly reflect adaptive plasticity rather than physiological malfunction, whereas most cases of central precocious puberty in boys are associated with brain abnormalities such as hypothalamic tumors and are unlikely to represent adaptive variation.^83^ A significant proportion of girls with premature adrenarche progress rapidly to menarche.^27^ Early adrenarche and menarche are both associated with prenatal growth restriction followed by rapid postnatal growth,^84^, ^85^ as well as increased adiposity and hyperinsulinemia in childhood.^86^ One possible interpretation is that adrenal and gonadal maturation are both accelerated in these girls as part of an alternative life‐history strategy entrained by prenatal nutrient restriction followed by ready postnatal availability of food. The rapid accumulation of fat and lean mass observed in these girls relative to their peers could, as opposed to the conventional view, which sees obesity as the cause of early puberty, be interpreted as a programmed preparation for early reproduction.

Early adrenarche has been considered a benign variant of normal development, but recent studies have suggested that early adrenarche predicts insulin resistance, metabolic syndrome, and polycystic ovary syndrome (PCOS) later in life.^82^, ^87^ But for the fact that PCOS is associated with reduced fertility, these associations could be considered costs of adaptation for early reproduction. The symptoms of PCOS, including high androgen levels, relative infertility, hirsutism, and android fat patterning, exemplify the plasticity of female maturation.^88^ Both cultural and genetic studies have identified PCOS as an ancient disorder, likely dating back 50,000 to 80,000 years.^89^ The high frequency of PCOS in the general population, which is up to 10% of women by NICHD criteria,^90^ has long been considered a challenge for evolutionary theory.^89^, ^91^, ^92^, ^93^, ^94^, ^95^, ^96^ The reduced fertility caused by this disorder results in women who are still able to reproduce, but with longer than average IBIs. For this reason, PCOS has been proposed to represent an alternative life‐history strategy in which affected women produce fewer but better provisioned offspring.^97^ A recent genetic study reported overtransmission of a risk allele to PCOS probands from heterozygous mothers but not from heterozygous fathers.^98^

Why should early puberty be more common in girls than boys? Is the pronounced sex difference in the frequency of precocious puberty related to the overall later maturation of boys than girls? Genome‐wide association studies find higher heritability and larger effect sizes for early pubertal timing in girls, but for late pubertal timing in boys,^37^ suggesting greater contributions of “normal” genetic variation to early maturation in girls but later maturation in boys. Within a group of small‐scale societies, Walker and colleagues found greater plasticity of female growth rates than male growth rates. They suggested that the sex difference might be explained by the exigencies of male–male competition.^80^ If young males have limited prospects for reproductive success because mating is dominated by older, larger, and more experienced males, then intrasexual competition may favor male maturation at older age and larger size.

## 7 CONCLUSION

The goal of this review has been to summarize current work in medicine, anthropology, psychology, and genetics that examines the complex transitions humans experience from birth to adulthood. We hope that we have also added something novel by exploring the likely influence that kin conflict has had on the evolutionary trajectory of our developmental pathway. Imprinted genes influence the timing of human weaning, adrenarche, and gonadarche. This suggests that the tempo of human development has been shaped by effects on the fitness of kin. While theories on developmental timing are beginning to recognize the importance of accounting for kin conflict,^99^ most models of the evolution of human life history attempt to predict how the timing of key events would maximize individual fitness. But natural selection is not predicted to maximize the fitness of individuals in the presence of intergenerational and intragenomic conflicts over costs and benefits. In particular, alleles of maternal and paternal origin are predicted to have partially antagonistic “goals.” Optimization models remain useful for defining the “goals” of particular actors, but the resolution of conflicts among actors will be determined not only by the actors' “goals” but also by their ability to influence outcomes. An understanding of the outcome of particular conflicts will require knowledge of both mechanisms (how) and functions (why). Current models of the evolution of human life history are beset by problems of too many degree of freedom because the values of key variables are not constrained by available data. An appreciation of the roles of imprinted genes in life‐history transitions and the directions of their effects will help to constrain the degrees of freedom of future models.
